# The influence of prenatal nutrition on ruminal microbiome of beef cattle

**DOI:** 10.3389/fgene.2025.1708543

**Published:** 2026-01-05

**Authors:** Édison Furlan, Guilherme Henrique Gebim Polizel, Arícia Christofaro Fernandes, Bárbara Carolina Teixeira Prati, Gabriela do Vale Pombo, Germán Darío Ramírez-Zamudio, Heidge Fukumasu, Miguel Henrique de Almeida Santana

**Affiliations:** Department of Animal Science, Faculty of Animal Science and Food Engineering, University of São Paulo, São Paulo, Brazil

**Keywords:** 16S rRNA metabarcoding, fetal programming, maternal nutrition, Nellore cattle, rumen

## Abstract

**Introduction:**

Maternal nutrition is recognized for inducing long-lasting effects on offspring performance during postnatal life. However, little is known about its potential role in modulating the ruminal microbiota during fetal development, as rumen colonization has traditionally been assumed to occur only at birth. This study aimed to evaluate the long-term effects of maternal nutrition during gestation on the offspring’s ruminal microbiota in postnatal life.

**Methods:**

The experimental design comprised 28 Nellore bulls, offspring of a single sire and born to primiparous heifers. Dams were assigned to two groups and received either mineral supplementation (Control; n = 14; 0.3 g/kg BW) or protein–energy supplementation (Supplemented; n = 14; 5 g/kg BW) throughout gestation, from conception to calving. Calves from both groups were managed identically from birth to slaughter. At the finishing phase, ruminal fluid samples were collected from 10 bulls per treatment. The V4 region of the 16S rRNA gene was sequenced, and amplicon sequence variants (ASVs) were identified using DADA2 and phyloseq for microbial diversity and taxonomic analysis. Pathway over-representation analysis was also conducted using MicrobiomeProfiler.

**Results and Discussion:**

Maternal nutrition resulted in modest yet significant alterations in the ruminal microbial communities of the offspring. The Supplemented group exhibited higher relative abundance of *Fibrobacter* and *Prevotellaceae UCG-003*, with reduced abundance of *Xylanibacter*. Pathway analysis revealed enrichment of starch and sucrose metabolism, along with modulation of amino acid biosynthesis and nitrogen metabolism, suggesting potential improvements in microbial protein synthesis and nitrogen utilization. In conclusion, maternal nutrition during gestation has long-term effects on the offspring’s ruminal microbiota, influencing specific bacterial taxa and metabolic pathways linked to carbohydrate metabolism and nutrient utilization.

## 1 Introduction

Maternal nutrition and other factors during pregnancy of cows have a significant impact on the offspring’s fetal development and postnatal growth. This phenomenon is referred to as “developmental programming” or “fetal programming” (FP) (Du et al., 2015). Studies in humans have shown a correlation between low birth weight and the development of metabolic disorders later in life (Barker, 1990; Godfrey and Barker, 2001), highlighting the crucial role of the maternal environment in fetal development. In the field of ruminants, seminal research from the 1950s and 1960s pioneered the investigation of how maternal nutrition could affect offspring development and herd productivity, a phenomenon known as the “maternal effect” (Short, 1955).

The impact of maternal nutrition and the prenatal environment on performance and meat quality of beef cattle have been extensively documented (Du et al., 2015; Maresca et al., 2019; Underwood et al., 2010). However, limited research has explored the impact of these factors on the development of the gastrointestinal microbiota, which plays a crucial role in metabolism and feed efficiency in beef cattle offspring (Amat et al., 2023). Studies conducted in mice have demonstrated that maternal gut microbiota can influence the metabolic conditioning of offspring from the embryonic stage (Jašarević and Bale, 2019). Evidence indicates that propionate generated by the maternal microbiota crosses the placenta and affects insulin levels in the developing embryo (Kimura et al., 1979). Additionally, restrictive maternal diets during pregnancy resulted in offspring with lower microbial diversity, associated with increased susceptibility to intestinal inflammation and metabolic disorders (Paz et al., 2024). A similar association has been observed between high-fat maternal diets and significant changes in the offspring’s gut microbiome composition, including a reduction in beneficial bacteria such as *Bifidobacterium* spp. and an increase in *Firmicutes* (Peng et al., 2023). These changes may contribute to an increased risk of obesity in offspring (Cook and Mansuy-Aubert, 2022). While these findings suggest a direct influence of the maternal gut microbiota on offspring development and metabolism during cow pregnancy, the precise mechanisms underlying this effect remain to be elucidated.

The ruminal microbiota, a densely populated microbial community that plays a direct role in nutrient metabolism, is a critical target for interventions aimed at enhancing animal productivity (Liu et al., 2021). Traditionally, nutritional strategies designed to modulate microbial composition have been applied predominantly during the growing and finishing phases, a framework sometimes referred to as “microbial programming” (Yáñez-Ruiz et al., 2015; Powell et al., 2018). Nevertheless, recent evidence suggests that this colonization may, in fact, begin *in utero* (Amat et al., 2021; Guzman et al., 2020). Consequently, the long-term impact of maternal nutrition on the establishment and functional maturation of the rumen microbiome remains largely unexplored. Understanding how maternal dietary factors influence microbial colonization during gestation and early life could reveal novel opportunities to improve offspring health, feed efficiency, and productivity through early-life microbial programming.

Based on that, it is hypothesized that maternal nutrition during pregnancy may cause changes in the ruminal microbiota of beef cattle offspring with long-term impacts throughout postnatal life. The objective of this study was to evaluate the long-term impact of prenatal nutrition on the ruminal microbiota of Nellore offspring up to the pre-slaughter stage.

## 2 Materials and methods

### 2.1 Experimental design and prenatal management

The present study was approved by the Research Ethics Committee of the Faculty of Animal Science and Food Engineering at the University of São Paulo (FZEA/USP) under protocol number 1843241117, in accordance with the guidelines of the National Council for the Control of Animal Experimentation (CONCEA).

A total of 28 young Nellore bulls, born from nulliparous heifers that underwent artificial insemination at a fixed time with sexed semen from a single Nellore bull, were subjected to two nutritional treatments during gestation. The heifers were randomly assigned to the treatments based on body weight (BW) and body condition score (BCS). The treatments consisted of mineral supplementation (0.3 g/kg of BW; Control–CON; n = 14) or protein-energy supplementation (5 g/kg of BW; Supplemented–SUP; n = 14). During the gestation period, the heifers were allocated to separate paddocks according to the treatment, with *Urochloa brizantha* cv. Marandu pasture (Table 1), and received the mineral or protein-energy supplement once daily at 1:00 p.m. (UTC−03:00; Table 2).

**TABLE 1:** Bromatological characterization of the pasture during the study.

Forage nutrients	CON	SUP
Dry matter (DM) (%)	91.8 ± 1.5	90.9 ± 1.2
Crude protein (CP) (%)	7.42 ± 0.70	7.53 ± 0.81
Ethereal extract (EE) (%)	1.87 ± 0.01	1.86 ± 0.01
Total digestible nutrients (TDN) (%)	60.2 ± 0.59	60.5 ± 0.64
Neutral detergent fiber (NDF) (%)	61.0 ± 1.49	62.4 ± 2.61
Calcium (%)	0.38 ± 0.04	0.39 ± 0.05
Phosphorus (%)	0.19 ± 0.01	0.20 ± 0.01

CON, Control; SUP, Protein-energy supplementation.

**TABLE 2:** Nutritional composition and ingredient formulation of the mineral and protein-energy supplements for the heifers.

Ingredients	Mineral supplement	Protein-energy supplement
Corn (%)	35.00	60.00
Soybean meal (%)	-	30.00
Dicalcium phosphate (%)	10.00	-
Urea 45% (%)	-	2.50
Salt (%)	30.00	5.00
Minerthal 160 MD (%)^a^	25.00	2.50
Nutrients		
Total digestible nutrients (TDN) (%)	26.76	67.55
Crude protein (CP) (%)	2.79	24.78
Non-protein nitrogen (%)	-	7.03
Acid detergent fiber (%)	1.25	4.76
Neutral detergent fiber (%)	4.29	11.24
Fat (%)	1.26	2.61
Calcium (g/kg)	74.11	6.20
Phosphorus (g/kg)	59.38	7.24

^a^ Mineral premix composition (Minerthal company, São Paulo, Brazil): Calcium = 8.6 g/kg; Cobalt = 6.4 mg/kg; Copper = 108 mg/kg; Sulfur = 2.4 g/kg; Fluorine = 64 mg/kg; Phosphorus = 6.4 g/kg; Iodine = 5.4 mg/kg; Manganese = 108 mg/kg; Selenium = 3.2 mg/kg; Zinc = 324 mg/kg; Sodium monensin = 160 mg/kg.

### 2.2 Postnatal management

Subsequent to parturition, the cows and their offspring were maintained in a single Marandu grass pasture (*Urochloa brizantha* cv. Marandu), wherein they had access *ad libitum* to mineral supplements until weaning, which occurred at 8 months of age. Subsequent to weaning, the calves were raised in a pasture setting for a period of 12 months, during which they were supplemented with an energy concentrate on a dry matter basis (30.19% of CP and 65.80% of TDN), receiving 3 g/kg of BW during the dry season, and with a protein supplement (30.04% of CP and 56.80% of TDN), receiving 1 g/kg of BW during the rainy season.

The finishing phase was conducted in a feedlot, with the bulls housed in individual pens measuring 12 m^2^ for a total period of 97 days. Prior to this, the animals were weighed after a 24-h fasting period and underwent a 15-day adaptation period to the facilities and diet, until they reached the final diet intake, which consisted of corn silage and concentrate in a 30:70 ratio (13.50% of CP and 75.40% of TDN; Table 3).

**TABLE 3:** Composition of the diets offered to the bulls during the finishing phase (finishing diet 90 days).

Ingredient	% DM diet	DMI (kg/day)
Corn silage	30.0%	3.54
Corn grain	61.7%	7.30
Soybean meal	5.00%	0.59
Urea	1.00%	0.12
Limestone	1.00%	0.12
White salt	0.50%	0.06
Minerthal 160 MD^a^	0.70%	0.09

^a^ Mineral premix composition (Minerthal company, São Paulo, Brazil): Calcium = 8.6 g/kg; Cobalt = 6.4 mg/kg; Copper = 108 mg/kg; Sulfur = 2.4 g/kg; Fluorine = 64 mg/kg; Phosphorus = 6.4 g/kg; Iodine = 5.4 mg/kg; Manganese = 108 mg/kg; Selenium = 3.2 mg/kg; Zinc = 324 mg/kg; Sodium monensin = 160 mg/kg; DM, dry matter; DMI, dry matter intake.

### 2.3 Slaughter and sample collection

Following a 97-day period of feedlot, the bulls were slaughtered at the Campus Fernando Costa slaughterhouse, situated 500 m from the feedlot, in the city of Pirassununga, São Paulo State, Brazil. Prior to slaughter, the bulls were stunned with a percussive bolt and then subjected to the jugular vein incision for bleeding, in accordance with the humane slaughter practices outlined in the Regulation for Health Inspection and Industrialization of Animal-Origin Products (Brasil, 2017).

The BW and ultrasound carcass traits collected at pre-slaughter stage (22 ± 2 months of age) are presented in Table 4. Further details regarding phenotypic measurements and methodology are provided in Pombo et al. (2025). Briefly, all phenotypic evaluations were analyzed using linear models in R (function “lm”), with treatment as a fixed effect; residuals were checked for normality (Shapiro–Wilk test) and homoscedasticity (Levene’s test).

**TABLE 4:** Ultrasound evaluation and body weight of calves at slaughter under different maternal nutrition strategies.

Trait	CON	SUP	*p*-value
Pre-slaughter BW (kg)	568.3 ± 54.7	564.6 ± 57.5	0.80
LMA (cm^2^)	114.58 ± 7.52	113.14 ± 8.19	0.59
SFT (mm)	10.41 ± 3.46	12.16 ± 3.18	0.18
RFT (mm)	7.92 ± 3.34	9.48 ± 3.55	0.24

The data are presented as mean ± standard error of the mean for loin muscle area (LMA), subcutaneous fat thickness (SFT), rump fat thickness (RFT), and body weight (BW) (Pombo et al., 2025).

Ruminal samples were collected from all slaughtered animals, but only ten bulls per treatment group were randomly chosen for 16S rRNA sequencing. Following the removal of the head, legs, skin, and viscera, the cranial ventral sac of the rumen was identified, and a sample of approximately 120 mL of ruminal fluid was collected within approximately 10 min. The material was then filtered through four layers of gauze, and an aliquot of approximately 5 mL was transferred to RNase/DNase-free cryogenic tubes for subsequent analysis.

### 2.4 16S rRNA gene sequencing and bioinformatics analyses

Genomic DNA was extracted from ruminal fluid samples using the PureLink™ Microbiome DNA Purification Kit (Invitrogen, Thermo Fisher Scientific, Waltham, MA, United States) following the manufacturer’s instructions. DNA quality and concentration were assessed using a Nanodrop One/One spectrophotometer (Thermo Fisher Scientific, United States) and agarose gel electrophoresis.

Sequencing of the V4 region of the 16S rRNA gene was performed on the Illumina iSeq platform with paired-end reads of 2 × 150 bp. The sequencing protocol involved two PCR steps. In the first PCR, the targeted V4 region was amplified from the template DNA, incorporating the Illumina overhang adapter sequences. The primers used were 515F (5′-GTGYCAGCMGCCGCGGTAA-3′) as forward and 806R (5′-GGACTACNVGGGTWTCTAAT-3′) as reverse, as described by Parada et al. (2016). PCR products were purified using MagBio HighPrep PCR beads, and fragment sizes were verified via agarose gel electrophoresis.

In the second PCR, sample-specific barcodes were added using the Nextera XT V2 kit, followed by additional purification, library validation with Agilent Bioanalyzer, and quantification using the Qubit dsDNA HS Assay (Life Technologies, United States). Libraries were pooled in equimolar amounts, and a heterogeneous control, phi-X phage, was added to the pool. Finally, the libraries and phi-X control were denatured and prepared for sequencing.

Sequence variants, designated as amplicon sequence variants (ASVs), were inferred using DADA2 v.1.32.0 and organized with phyloseq v.1.48.0, as described by Callahan et al. (2016) and McMurdie and Holmes (2013).

Briefly, raw sequencing reads were processed using DADA2 v.1.32.0 in R v.4.4.0, filtered to remove low-quality bases (Q ≥ 30) and adapter contamination, dereplicated, merged, and filtered to remove PCR-related artifacts and PhiX-related chimeras. Taxonomic annotation was performed using the SILVA non-redundant database (v.138.2, nr99). ASV abundance tables, taxonomic assignments, and sample metadata (CON and SUP groups) were structured as phyloseq objects. Phyla with only a single feature and ASVs with prevalence <15% (present in fewer than three samples) were removed prior to downstream analysis. Alpha diversity indices were calculated to examine the influence of FP on the ruminal microbiota. Differences between groups were tested using linear models, considering only treatment as a fixed effect, with residuals evaluated for normality (Shapiro–Wilk test) and homoscedasticity (Levene test). Beta diversity was analyzed via Principal Coordinates Analysis (PCoA) using log_2_(x+1)-transformed ASV counts to identify clustering patterns among samples. Differential abundance was assessed with DESeq2 v.1.44.0. ASVs with adjusted *p*-value <0.05 were considered differentially abundant. Additionally, we performed a correlation analysis of the phenotypes in Table 4 with the differentially abundant ASVs using Spearman’s test (p < 0.05) via the cor() function in the R statistical environment.

Functional enrichment was performed on differentially abundant ASVs. Nucleotide sequences were translated into amino acids using EMBOSS Transeq and annotated with the GhostKOALA database (genus_prokaryotes + viruses) to assign KEGG Orthology (KOs) (Polizel et al., 2025). Overrepresentation analysis was conducted using MicrobiomeProfiler v.1.10.0 to identify metabolic pathways associated with the differentially abundant ASVs. Pathways with false discovery rate (FDR) <0.1 were considered significant.

## 3 Results

### 3.1 Alpha and beta diversity

**FIGURE 1:**
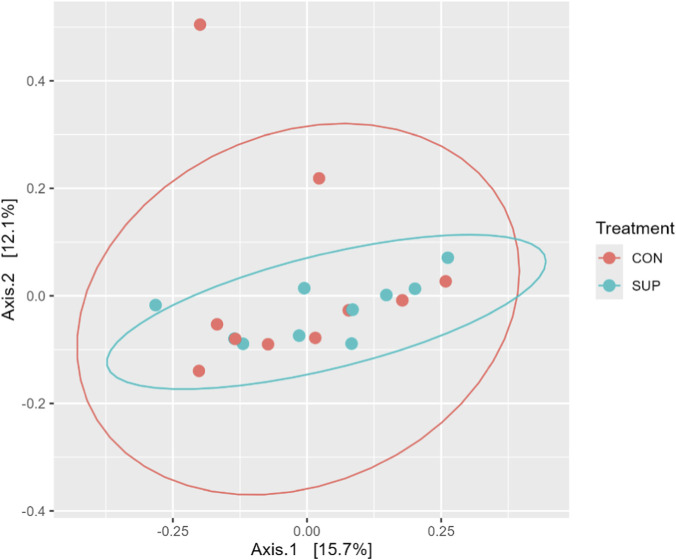
Principal coordinate analysis (PCoA) of the ruminal microbiota composition.

The PCoA (Figure 1) revealed substantial overlap between the CON and SUP groups, suggesting no major differences in microbial community structure between treatments. The first and second axes explained 15.7% and 12.1% of the total dissimilarity, respectively.

Alpha diversity analyses indicated that prenatal nutrition had no significant effect on the ruminal microbiota composition in the progeny. The Observed (p = 0.33), Shannon (p = 0.44), Simpson (p = 0.45), and Fisher (p = 0.39) indices are shown in Table 5.

**TABLE 5:** Alpha diversity indices of the microbiota from Nellore bulls with different prenatal nutrition.

Alpha diversity	CON	SUP	*p*-value
Observed	621.2 ± 114.0	681.5 ± 152.0	0.33
Shannon	4.833 ± 0.498	4.978 ± 0.296	0.44
Simpson	0.966 ± 0.025	0.973 ± 0.012	0.45
Fisher	97.90 ± 19.24	106.2 ± 22.77	0.39

CON, Control; SUP, protein-energy supplementation.

### 3.2 Differentially abundant amplicon sequence variants (ASVs)

**FIGURE 2:**
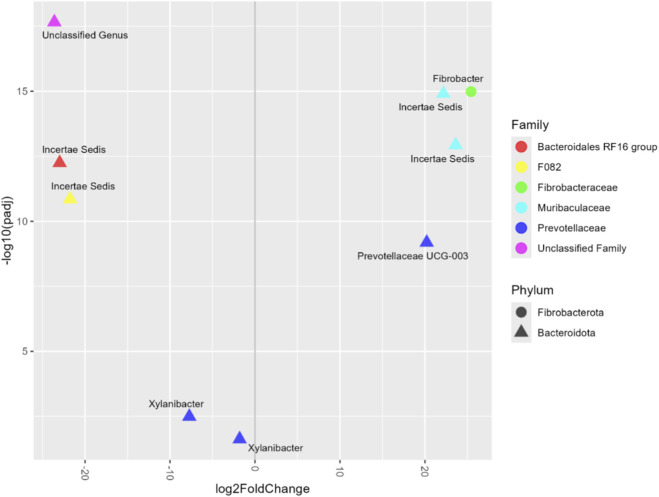
Volcano plot of differentially abundant bacterial taxa showing only significant amplicon sequence variants (ASVs). The X-axis represents the log2 fold change, and the Y-axis represents the transformed statistical significance (-log10 of the adjusted p-value, padj). Positive values on the X-axis indicate higher abundance in the supplemented group (SUP).

A volcano plot is employed to showcase the differential abundance of various bacterial taxa between groups, with only the significant ASVs (FDR <0.05) being displayed (Figure 2). Statistical information on the significant ASVs can be found in Table 6.

**TABLE 6:** Differentially abundant amplicon sequence variants (ASVs) identified in the rumen of Nellore bulls from different prenatal nutrition groups (Con and SUP).

ASVs	Phylum	Class	Order	Family	Genus	log2 fold change	*padj*
ASV698	*Bacteroidota*	*Bacteroidia*	*Sphingobacteriales*	*Unclassified family*	*Unclassified genus*	−23.61	2.20e^−18^
ASV64	*Fibrobacterota*	*Fibrobacteria*	*Fibrobacterales*	*Fibrobacteraceae*	*Fibrobacter*	25.42	1.03e^−15^
ASV434	*Bacteroidota*	*Bacteroidia*	*Bacteroidales*	*Muribaculaceae*	*Incertae Sedis*	22.16	1.23e^−15^
ASV205	*Bacteroidota*	*Bacteroidia*	*Bacteroidales*	*Muribaculaceae*	*Incertae Sedis*	23.61	1.18e^−13^
ASV128	*Bacteroidota*	*Bacteroidia*	*Bacteroidales*	*Bacteroidales RF16 group*	*Incertae Sedis*	−22.99	5.69e^−13^
ASV250	*Bacteroidota*	*Bacteroidia*	*Bacteroidales*	*F082*	*Incertae Sedis*	−21.77	1.39e^−11^
ASV481	*Bacteroidota*	*Bacteroidia*	*Bacteroidales*	*Prevotellaceae*	*PrevotellaceaeUCG-003*	20.20	6.57e^−10^
ASV538	*Bacteroidota*	*Bacteroidia*	*Bacteroidales*	*Prevotellaceae*	*Xylanibacter*	−7.72	0.003
ASV40	*Bacteroidota*	*Bacteroidia*	*Bacteroidales*	*Prevotellaceae*	*Xylanibacter*	−1.80	0.023

The ASVs ASV538 and ASV40 (genus *Xylanibacter*) were more abundant in the CON group, whereas ASV434 and ASV205 (family *Muribaculaceae*) were more abundant in the SUP group. Other ASVs also differed between groups, including ASV481 (genus *Prevotellaceae UCG-003*), ASV250 (family *F082*), and ASV128 (family *Bacteroidales RF16 group*). Additionally, ASV64 (genus *Fibrobacter*) was more abundant in the SUP group.

In the table below (Table 7), we have included the results of significant correlations between ASVs and phenotypes. Significant correlations (p < 0.05) were only found for the ASVs in the CON group.

**TABLE 7:** Significant spearman correlations of abundant ASVs with phenotypes of Nellore bulls in the control group (CON).

ASVs	Phenotype	r	*p*-value
ASV128	SFT	−0.69	0.028
ASV128	RFT	−0.68	0.029

ASVs, amplicon sequence variants; SFT, subcutaneous fat thickness; RFT, rump fat thickness.

### 3.3 Overrepresentation analysis (ORA)

**FIGURE 3:**
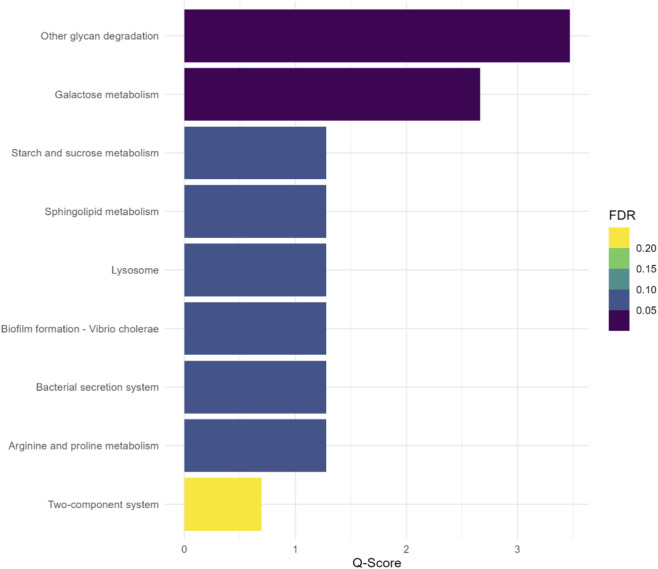
Enriched metabolic pathways based on the differentially abundant amplicon sequence variants (ASVs).

The ORA revealed eight significantly enriched metabolic pathways associated with the differentially abundant ASVs (Figure 3). These included other glycan degradation (FDR = 0.00034), galactose metabolism (FDR = 0.00216), starch and sucrose metabolism (FDR = 0.05262), sphingolipid metabolism (FDR = 0.05262), lysosome (FDR = 0.05262), biofilm formation–*Vibrio cholerae* (FDR = 0.05262), bacterial secretion system (FDR = 0.05262), and arginine and proline metabolism (FDR = 0.05262). The two-component system pathway was not significantly enriched (FDR = 0.20116).

## 4 Discussion

The findings of the present study provide evidence supporting the hypothesis that maternal nutrition may influence the ruminal microbiota of the offspring, which could have potential implications for nutritional management strategies and the long-term improvement of productive efficiency in beef cattle (Li et al., 2019; Kolathingal-Thodika et al., 2025). Studies indicate that the initial rumen microbiota of the calf reflects that of the mother, and this similarity can be observed until the weaning period (Barden et al., 2020). Nonetheless, our findings indicate that a subtle maternal influence can still be detected during the finishing phase.

The idea of modulating the microbiota of animals in the earliest stages of life emerges as an interesting strategy to improve nutrient utilization efficiency. As a consequence of this improved utilization, we can mention the improvement of environmental sustainability through the reduction of greenhouse gas emissions. The metabolic adaptations promoted by fetal programming in the host and its microbiome, unlike the use of feed additives to mitigate methane emissions, present themselves as a functional long-term strategy (Kolathingal-Thodika et al., 2025).

Among the differentially abundant ASVs identified, the genus *Fibrobacter* is recognized for its capacity to degrade cellulose in the rumen (Gharechahi et al., 2021). This activity is crucial for the release of fermentable sugars, resulting in the production of VFAs, which are the primary energy source for ruminants (Ungerfeld, 2020). Additionally, members of the *Prevotellaceae* family, particularly the UCG-003 group, are associated with the fermentation of complex polysaccharides and proteins, contributing to the production of propionate, a VFA that is essential for hepatic gluconeogenesis (Betancur-Murillo et al., 2022). Furthermore, *Bacteroidota* and *Fibrobacterota*, even when not classified at the genus level, are known for their metabolic versatility in the degradation of structural polysaccharides, playing a key role in rumen digestive efficiency (Chen et al., 2024). Taken together, these observations potentially indicate that the ASVs identified not only contribute to carbohydrate and protein metabolism but also to ruminal homeostasis, microbial resilience, and host performance (Stewart et al., 2018). In the context of intensive rearing and supplementation strategies, the activity of these ASVs may explain differences in growth, feed efficiency, and muscle development, suggesting that the ruminal microbiome mediates the interaction between diet, microbial function, and productive outcomes in beef cattle (Andrade et al., 2022). Our study showed few correlations between phenotypes and ASVs, with only one ASV (*Bacteroidales RF16 group* Family) being significant for carcass fat deposition traits (SFT and RFT). Although our previous study (Pombo et al., 2025) showed no phenotypic differences at the finishing phase, some differences were observed before weaning. These results indicate that, even if phenotypic differences diminish over time, changes in the ruminal microbiome and associated metabolic pathways may have potential long-lasting effects.

Regarding the overrepresentation analysis, the enrichment of the glycan degradation pathway indicates a strong microbial potential to break down complex polysaccharides into fermentable sugars, which likely enhances the production of volatile fatty acids (VFAs) (Seshadri et al., 2018). The *Prevotellaceae* family is related to glycan metabolism in cattle, including its degradation (Betancur-Murillo et al., 2023). Three significant ASVs from this family were found, and these can be related to at least two main enriched pathways (glycan and starch metabolism) (Shinkai et al., 2024).

Similarly, the galactose, starch, and sucrose metabolism pathways suggest that these microorganisms can efficiently convert simple and complex carbohydrates into bioavailable energy, optimizing nutrient utilization depending on feed composition and potentially improving weight gain and energy balance (Stewart et al., 2018). *Fibrobacter* (ASV64) is directly related to the metabolism of cellulolytic carbohydrates (Forano et al., 2008). This type of microorganism has already been linked to the metabolism of galactose, starch, and sucrose (Neumann et al., 2018; Fakih et al., 2023), which were also identified in the enriched pathways in this study. Those processes provide a primary energy source for ruminants, potentially supporting higher growth rates, improved feed conversion, and greater energy availability for physiological functions such as maintenance, muscle accretion, and thermoregulation (Bergman, 1990).

The arginine and proline metabolism pathway highlights a potential contribution of these ASVs to protein synthesis and nitrogen cycling, providing precursors for muscle accretion and influencing overall growth performance (Fung et al., 2025). Pathways related to sphingolipid metabolism and lysosomal activity may support the maintenance of cellular and membrane integrity, promote ruminal epithelial health and contribute to the resilience of the microbial community (Zhao et al., 2023). Sphingolipid metabolism is related to a series of enzymes that target kinases, phosphatases, lipases, membrane receptors, among others (Hannun and Obeid, 2018). The relationship between these bioactive lipids and ASV698 was demonstrated in a study on sheep, where enriched pathways of sphingolipid metabolism in animals were also linked to *Sphingobacteriales* (Li et al., 2025). Additionally, biofilm formation and bacterial secretion systems suggest mechanisms by which these ASVs enhance microbial colonization, community stability, and interactions with the host, potentially increasing resistance to dietary shifts or ruminal disturbances (McAllister et al., 1994). Despite changes in the ruminal microbiota, in terms of phenotypic performance there were no significant changes in live weight or pre-slaughter carcass traits. Possibly, the molecular changes observed by the variation in the microbiome were not sufficient to alter these phenotypes.

A comprehensive understanding of these metabolic pathways is imperative for the optimization of management and supplementation strategies in livestock, with the objective of enhancing feed efficiency, sustainability and herd health. Although much of our discussion is based on potential implications rather than direct experimental evidence, this understanding serves as a foundational element for future research on the ruminal microbiota and its interactions with diet. Consequently, when formulating strategies for beef cattle management, it is important to adopt a holistic approach that considers not only the immediate health of the animals but also the influence of current nutritional practices on the performance and health of future generations.

In summary, our findings indicate that maternal nutrition potentially exerts a measurable influence on the offspring ruminal microbiota, particularly affecting metabolic pathways related to carbohydrate degradation and energy-yielding fermentation, possibly guided by the epigenetic effects promoted by the higher concentration of propionate ingested by cows (fetal programming). These results emphasize the rumen as a biologically plastic system during early developmental windows and underscore the importance of maternal management in shaping digestive efficiency trajectories later in life.

We acknowledge several limitations in the current study, including the relatively small sample size. Increasing the number of animals in future studies could provide greater statistical power and enable the detection of more subtle effects of maternal nutrition on offspring ruminal microbiota. Furthermore, longitudinal studies evaluating multiple time points are needed to determine whether the differences in microbiome composition between prenatal nutrition groups persist, diminish, or evolve over time. Integrating multi-omics approaches, such as metagenomics and metabolomics, would offer more comprehensive insights into the functional consequences of maternal dietary interventions. Expanding the diversity of animal populations, including different breeds or management systems, could also enhance the generalizability of the findings.

Collectively, these approaches would refine our understanding of how prenatal nutrition shapes offspring microbial communities and inform strategies to optimize animal health, digestive efficiency, and productivity.

## 5 Conclusion

Maternal nutrition during gestation influenced specific bacterial taxa and metabolic pathways in the offspring’s ruminal microbiota, particularly those associated with carbohydrate metabolism and glycan degradation. These findings indicate a potential role of prenatal nutrition in modulating long-term ruminal function and nutrient utilization efficiency which may result in phenotypic changes such as fat deposition in the carcass in beef cattle offspring.

## Data Availability

The datasets presented in this article are not readily available due to ethical restrictions and the nature of the animal model used in this study. Requests to access the datasets should be directed to the corresponding authors, subject to approval from the relevant institutional review board (IRB), and in compliance with applicable regulations.
